# Sequence homology between HLA-bound cytomegalovirus and human peptides: A potential trigger for alloreactivity

**DOI:** 10.1371/journal.pone.0178763

**Published:** 2017-08-11

**Authors:** Charles E. Hall, Vishal N. Koparde, Maximilian Jameson-Lee, Abdelrhman G. Elnasseh, Allison F. Scalora, David J. Kobulnicky, Myrna G. Serrano, Catherine H. Roberts, Gregory A. Buck, Michael C. Neale, Daniel E. Nixon, Amir A. Toor

**Affiliations:** 1 Bone Marrow Transplant Program, Massey Cancer Center, Virginia Commonwealth University, Richmond, Virginia, United States of America; 2 Center for the Study of Biological Complexity, Virginia Commonwealth University, Richmond, Virginia, United States of America; 3 Department of Microbiology and Immunology, Virginia Commonwealth University, Richmond, Virginia, United States of America; 4 Departments of Psychiatry and Human & Molecular Genetics, Virginia Commonwealth University, Richmond, Virginia, United States of America; 5 Division of Infectious Diseases, Virginia Commonwealth University, Richmond, Virginia, United States of America; Beth Israel Deaconess Medical Center, UNITED STATES

## Abstract

Human cytomegalovirus (hCMV) reactivation may often coincide with the development of graft-versus-host-disease (GVHD) in stem cell transplantation (SCT). Seventy seven SCT donor-recipient pairs (DRP) (HLA matched unrelated donor (MUD), n = 50; matched related donor (MRD), n = 27) underwent whole exome sequencing to identify single nucleotide polymorphisms (SNPs) generating alloreactive peptide libraries for each DRP (9-mer peptide-HLA complexes); Human CMV CROSS (Cross-Reactive Open Source Sequence) database was compiled from NCBI; HLA class I binding affinity for each DRPs HLA was calculated by NetMHCpan 2.8 and hCMV- derived 9-mers algorithmically compared to the alloreactive peptide-HLA complex libraries. Short consecutive (≥6) amino acid (AA) sequence homology matching hCMV to recipient peptides was considered for HLA-bound-peptide (IC50<500nM) cross reactivity. Of the 70,686 hCMV 9-mers contained within the hCMV CROSS database, an average of 29,658 matched the MRD DRP alloreactive peptides and 52,910 matched MUD DRP peptides (p<0.001). *In silico* analysis revealed multiple high affinity, immunogenic CMV-Human peptide matches (IC50<500 nM) expressed in GVHD-affected tissue-specific manner. hCMV+GVHD was found in 18 patients, 13 developing hCMV viremia before GVHD onset. Analysis of patients with GVHD identified potential cross reactive peptide expression within affected organs. We propose that hCMV peptide sequence homology with human alloreactive peptides may contribute to the pathophysiology of GVHD.

## Introduction

Human cytomegalovirus and other viral infections pose a significant hurdle to successful stem cell transplantation, affecting morbidity and mortality rates among the immunocompromised populations the world over. [1,2] Human CMV seropositivity has been estimated in 50–80% of the population in the United States by age 40. [3] While the majority of infected patients are asymptomatic due to viral latency after initial viral clearance, SCT patients with newly reconstituted immune systems exhibit rates of reactivation approximately 30–65% in multiple studies. [4–7] Furthermore, hCMV reactivation often coincides with the incidence of another serious complication of SCT, i.e., GVHD. [8–11] Although many groups have historically separated GVHD incidence from hCMV infection data, even by complete exclusion in clinical trial design, there has been growing evidence that these diseases are not mutually exclusive and are likely linked in a number of ways beyond treatment. [12,13]

GVHD pathophysiology is driven by donor T cell mediated alloreactivity, directed against recipient mHA. These are recipient-derived oligopeptides presented on HLA molecules, and result from coding nucleotide sequence variation. Whole exome sequencing has been used to identify the entire library of nucleotide variation that exists between the exomes of HLA matched SCT donors and recipients. [14] From these data it is possible to derive, *in silico*, all the *potential* unmodified alloreactive peptides which bind the relevant HLA (class I for example) in a donor-recipient-pair (DRP), and in turn this metric may be used to estimate a patient’s given potential to develop alloreactivity, or GVHD. [15] Aside from helping understand GVHD pathophysiology, this library of recipient-derived mHA-HLA complexes may also be used to interrogate the relationship between pathogen antigens, as well as tumor antigens, and the disease states that may result, in particular the development of cross-reactive illness, such as GVHD triggered by CMV or other viral reactivation.

**Fig 1:**
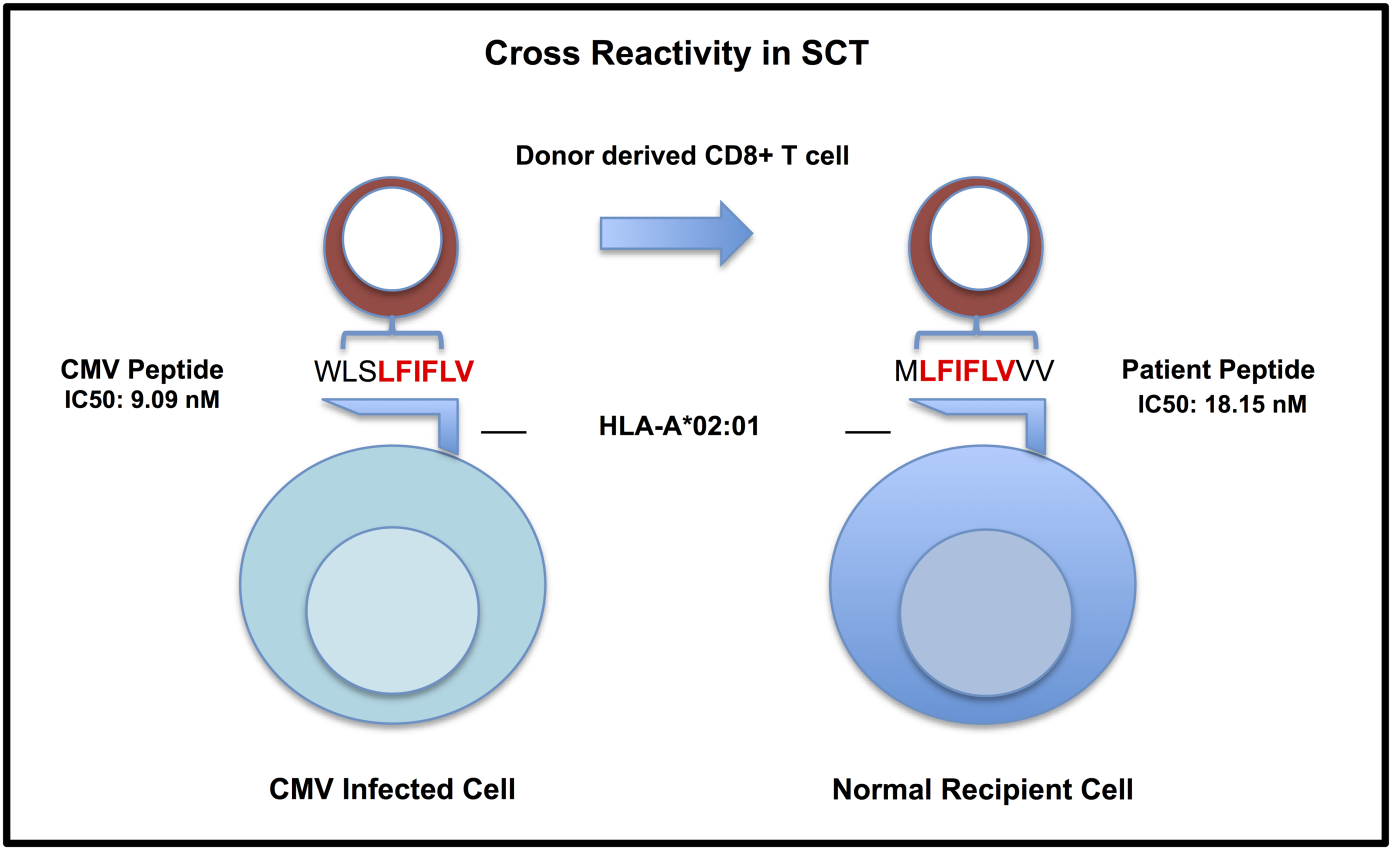
Hypothetical CMV—Human antigenic mimicry model. Proposed cross-reactivity model for CMV-specific T cells that may react to matched homologous peptides (≥6/6 consecutive amino acids) bound to the same HLA on normal recipient cells present in GVHD-affected tissues.

T cell cross reactivity originally was uncovered in relation to autoimmunity, especially in the context of CMV [16–19] and solid organ transplantation. [20–23] T cell cross reactivity occurs because, TCR-peptide-HLA class I bound complexes exhibit a strong recognition of 2–4 central amino acid residues in various orientations and allow for multiple amino acid substitution to the flanks in anchor positions while relying on HLA and peptide sequences simultaneously. The idea of T cells reacting to antigens with amino acid sequence homology (≥6/6 consecutive AA residues in a 9-mer for example) on different cell types with a given HLA-class I type has taken hold from multiple clinical examples. [17] Therefore, while CMV and other B-Herpesviruses have been shown to be associated with autoimmunity and contribute to oncogenic progression possibly affecting relapse rates, [24–26] CMV-specific T cell cross reactivity through adoptive lymphocyte transfer has been shown effective in the treatment of glioblastoma multiforme upon reactivation. [27,28] These clinical trials centering on confirmed CMV-specific T cell cross reactivity with infected tumor cells highlights the expectation of eliciting an immune response to CMV and ‘self’ tumor cells simultaneously. However, the development of CMV-specific T cells in that manner raises the question of eliciting a cross-reactive GVHD response against HLA bound-alloreactive recipient peptides. In theory, this may occur due to peptide polymorphisms, resulting from single nucleotide polymorphisms (SNPs). Resulting polymorphic peptides in the recipient with enough sequence homology to CMV peptides to elicit a HLA-specific immune response, may be presented as targets to donor lymphocytes (Fig 1).

Therefore, if CMV-derived HLA class I bound oligopeptide have sequence homology with human, alloreactive peptides bound to the same HLA class I and trigger cross recognition by donor T cells, the presence of these cross-reactive peptides may constitute a risk factor for the development of GVHD following SCT. In this study, HLA bound peptide-match analysis is facilitated by a newly compiled Human CMV CROSS (Cross-Reactivity Open Source Sequence) database. There were CMV—human peptide sequence matches present, and in a majority of CMV sero-positive patients with GVHD, this was corroborated by GVHD-specific organ incidence of the human peptide source genes.

## Methods

### Whole exome sequencing and peptide library creation

Patients with recurrent or high-risk hematological malignancies undergoing allogeneic SCT at Virginia Commonwealth University were included in this retrospective study following Virginia Commonwealth University (VCU) Institutional Review Board approval, MCC-14012 Study. VCU IRB waived the need for informed consent to MCC-14012 on all adult participant archived samples, de-identified by clinical research staff. The demographic and clinical data were not available to the exome sequencing team, and exome sequencing data was not available to the research team, with the exception of SNP identity in a donor recipient specific manner. Exome sequencing data are maintained on secure server with the VCU Center for the Study of Biological Complexity. Previously cryopreserved DNA samples were whole exome sequenced following de-identification by the BMT research team. These recipients received an allograft from either a related or an unrelated, HLA-matched donor at 8/8 or 7/8 HLA loci S1 Table. There were no significant differences in the HLA-matched and HLA-mismatched related and unrelated donors, therefore these were considered together. The transplant DRP, were annotated for identification of all the non-synonymous single nucleotide polymorphisms (nsSNPs), across the whole exome, in the graft versus host (GVH) direction (nsSNP(GVH)), i.e., polymorphism present in the recipient but absent in the donor. [14] Each nsSNP(GVH) was analyzed using the ANNOVAR software, [29] as previously described, [15] to populate the flanking amino acids around each amino acid coded for by each of the polymorphisms. This was accomplished through sequence padding, using DB SNP130 and hg18 genome coordinates, and yielded 17-mer peptides with variant amino acids occupying the central location. This 17-mer oligopeptide library created the opportunity to derive 9 separate 9-mer recipient oligopeptides, using a sliding window method, with the polymorphism-derived amino acid positioned at position 1–9 from C- to N-terminus. The nine oligopeptides generated per nsSNP(GVH) were extended to the whole exome for each recipient, representing each DRPs’ unique peptide library.

### *In silico* peptide-HLA binding affinity determination

Patient peptide libraries were initially analyzed by the NetMHCpan software, version 2.8. [30,31] The analytic output yielded source gene information, polymorphic peptide sequence (9-mer), and a calculated IC50 value from the NetMHCpan algorithms for each of the six HLA class I molecules, HLA-A, B and C in each DRP. IC50 values (nM) indicated the amount of peptide required to displace 50% of intended or standard peptides specific to a given HLA. Binding affinity is inversely related to IC50 values such that a smaller IC50 value indicated a stronger affinity. The variant alloreactive peptides with a cutoff value of IC50 ≤500 nM to the relevant HLA were included, and designated, *presented peptides*; and those that had an IC50 ≤50 nM, were termed *strongly presented* peptides. IC50 values in this range (IC50<500 nM) were considered relevant in previous studies of HLA-binding [32] and predictive mouse models of CTL response to vaccinia virus epitopes. [33] Further processing and interrogation of HLA binding in sliding 9-mer windows allowed for affinity sorting and HLA-specific separation along with gene information.

### Determining sequence homology between human and CMV derived HLA bound peptides

**Fig 2:**
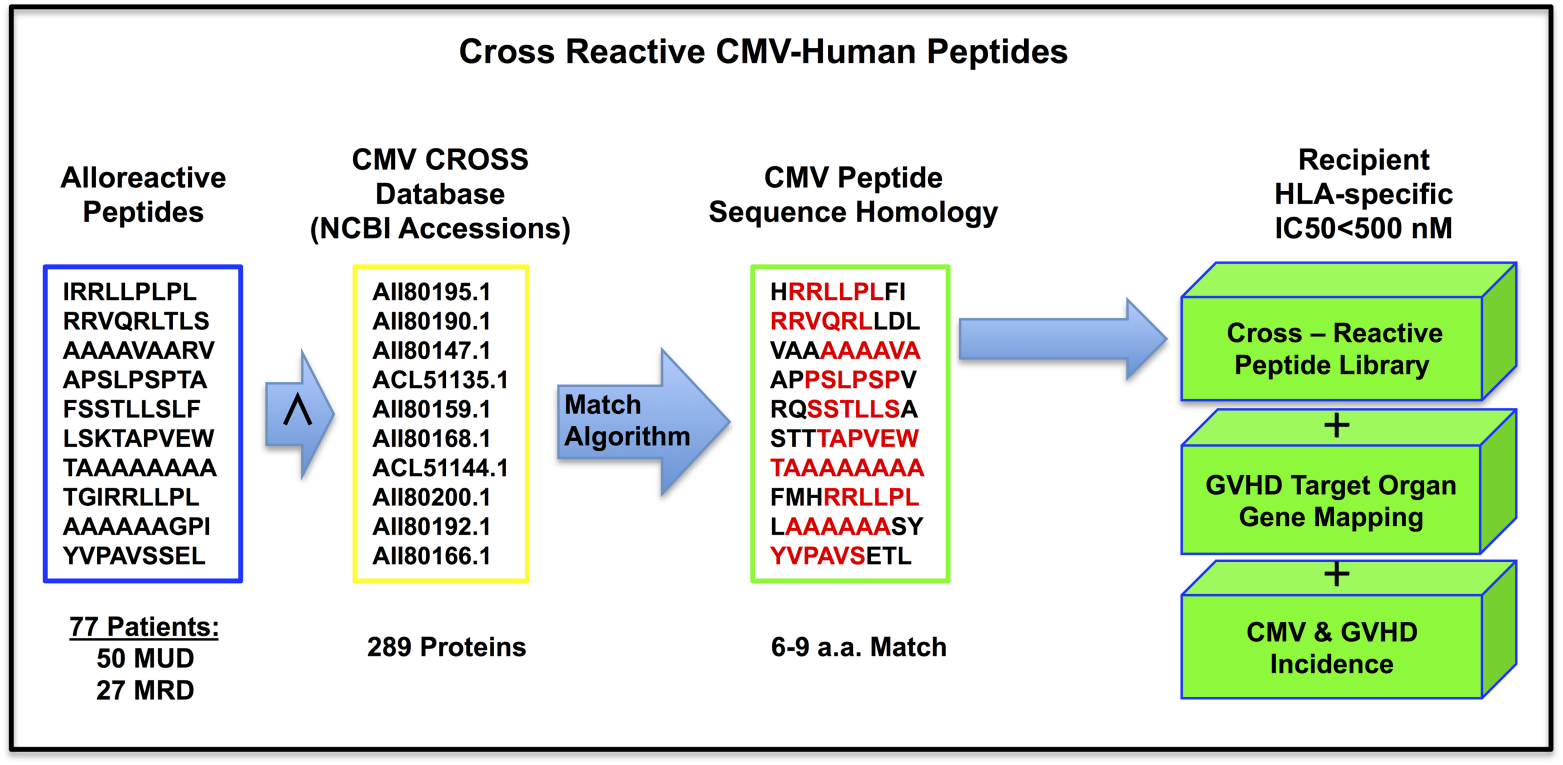
Bioinformatic pipeline of the alloreactive peptide library comparison with the CMV proteome. A determination of the potential for Pro-GVHD DRP cross-reactivity upon CMV reactivation correlated with GVHD-tissue specific distribution of peptides & immunogenicity.

The bioinformatic pipeline utilized to interrogate SCT DRPs for HLA bound polymorphic peptides derived from nsSNP(GVH) in each DRP was extended and refined to interrogate each SCT DRP for relevant HLA class I bound CMV peptides. The binding of CMV-derived peptides to specific HLA Class I molecules was the first step towards determining their sequence homology with human HLA bound peptides. This bioinformatic pipeline is depicted in Fig 2. The initial step was *compilation* of the Human CMV-CROSS (Cross-Reactivity of Open Source Sequences). This is a database of 289 hCMV proteins or variants representative of the entire known multi-strain inclusive (Merlin, Towne, Toledo and AD169) hCMV proteome sourced from NCBI S1 File (Protein variants had a single or multiple amino acid difference reported for the same protein/CMV gene; Deduplication was performed to remove bias). [34] The next step was the *utilization* of previously created patient polymorphic peptide libraries (derived from nsSNP(GVH) in each DRP) for initial feasibility of BLAST protein sequence alignment analysis. [35] This was followed by a subsequent confirmatory CMV-Human sequence homology analysis. Sequence homology relied on a match of 6 or more continuous amino acids in a string of 9 total amino acids (6/6-9/9) by sliding window analysis to identify sequence overlap between HLA bound 9-mer alloreactive human and hCMV peptides. Next, HLA Class I binding prediction (NetMHCpan) was performed for the hCMV peptides, screening the generated hCMV 9-mer peptides for HLA binding affinity across the test patient population (n = 9).

A specific streamlined bioinformatics application (Table 1) was developed to directly compare the alloreactive donor-recipient peptide libraries for all the patients (n = 77) to the HLA bound hCMV peptides by sequence homology; eliminating the need for the Protein BLAST sequence alignment (≥6/6 match by 9-mer sliding window analysis using all 9-mers extracted from each database, DRP peptide library or Human CMV CROSS proteome, pre- binding affinity screening). Binding affinity was predicted (NetMHCpan) for all the 70,686 hCMV 9-mer peptides (resulting from the 289 hCMV proteins) to the known 2,915 human HLA-class I types along with collection of source gene information (hCMV and Human geneids), which were used to sort the peptides for strength of binding affinity. Immunogenicity screening was then performed to validate CMV-human matched peptides for the presence of hCMV gene products previously reported as CD8+ T cell targets known to elicit specific immune responses.

**Table 1:** HCMV cross-reactivity potential of 77 SCT DRP from whole exome sequencing data using sliding window match analysis.

**1**	**289 HCMV protein sequences representative of the HCMV proteome were downloaded from NCBI by batchentrez utility**
**2**	**Downloaded CMV protein sequences were sliced into 9AA peptides and deduplicated, resulting in a library of 70686 9AA peptides**
**3**	**Each of the 70686 9AA peptides were further divided into 2 8AA, 3 7AA and 4 6AA peptides to form a lookup table for each component peptide (6,7,8) with the one or more 9AA parent peptide(s) and the associated CMV geneid**
**4**	**As a focus on Class I HLA, a list of the 2915 plausible HLA-A/B/C types interrogated by netmhcpan was compiled**
**5**	**IC50 values for all 70686 9AA CMV peptides were precalculated against all 2915 HLA types and saved in the CROSS database**
**6**	**9-mer peptides isolated from ANNOVAR per patient were searched for match with the 9AA CMV peptide pool** **… if no match found then each 9-mer was split into 2 8-mers and each searched in the 8AA CMV peptide pool** **… if still no match found then each 9-mer was split into 3 7-mers and each searched in the 7AA CMV peptide pool** **… if still no match found then each 9-mer was split into 4 6-mers and each searched in the 6AA CMV peptide pool**
**7**	**If any of the above searches found a match, the search was terminated and results for that patient 9-mer peptide reported**
**8**	**Reported 9-mers were linked with geneid (Human & CMV), HLA type, IC50 (Human & CMV), partial peptide (6/7/8), CMV_peptide (9)**
**9**	**Each patient-specific peptide library generated by comparing alloreactive peptides with the CROSS database were compiled, statistically analyzed (Student's T-Test) and further screened for high affinity binding (1/IC50 or an IC50 range of 0–500 nM)**
**10**	**Binding strength (IC50<500 nM) of homologous CMV and human peptides to DRP-specific HLA was compared for pattern analysis**
**11**	**Immunogenicity of common CMV gene products as CD8+ T cell targets was screened for validation on CMV infected patients (n = 30)**
**12**	**CMV peptide matches to Human alloreactive peptides underwent multiple rounds of deduplication to ensure only unique peptide inclusion by both software programming (Excel or independent coding) and finally by human observation upon final analysis**.
**13**	**Gene Tissue Expression (GTEx) data was gathered on CMV infected patients (n = 30) to find GVHD specific organ incidence**
**14**	**Case studies were prepared, 6 patients with CMV before GVHD: 2 acute GVHD, 2 chronic GVHD and 2 aGVHD + cGVHD**.

### Tissue distribution of cross-reactive peptides

Homologous CMV-Human peptide libraries were compiled for each DRP and the source genes organized for putative GVHD target tissue-specific distribution analysis using the GTEx portal for expression data. [37] Each patient with GVHD involving specific tissues (e.g., skin) had tabulation of those disease-incident tissue-specific alloreactive peptides presented by their HLA (e.g., 10 skin peptides from 8 genes expressed in the skin) and the remainder GVHD tissue peptides/genes were tabulated as other or nonspecific to the patient’s disease then tallied. Following criteria were used to assign CMV-Human peptide cross-reactivity potential: 1. Amino acid sequence homology of 6/6 to 9/9 (9-mers) between CMV and Human HLA bound peptides 2. IC50 values less than 500 nM for binding to the unique HLA in each DRP (Presented or strongly presented peptides) 3. Human Gene-GVHD Tissue Expression Connection (Patient specific, i.e. skin, GI, etc.)

### Patients

CMV reactivation defined as ≥200 copies of hCMV DNA/μL of plasma was determined by quantitative polymerase chain reaction. Absolute lymphocyte counts were measured at least twice weekly following SCT during the first 100 days and at least once weekly out to 6 months following SCT. Human CMV titers, lymphocyte counts and serum immunosuppressant drug levels were collected to one year post SCT per MCC-14012 Study. Acute and chronic GVHD was diagnosed according to Glucksberg and NIH 2005 Consensus clinical criteria, respectively. Lymphoid recovery patterning was assessed to examine differences among patients with CMV alone, GVHD alone, both or neither conditions post SCT. Three patterns were observed as previously described [38]: Pattern A or early (ALC ≥ 1 Billion cells/L by Day 45 post SCT), Pattern B or middle (ALC ≥ 0.5 up to <1 Billion cells/L by Day 45 post SCT), and Pattern C (ALC <0.5 Billion cells/L by Day 45 post SCT). Student’s T test and Pearson correlation were utilized for statistical analysis of the alloreactive/CMV peptide data presented. Cox regression hazard ratio and Kaplan-Meier Survival were utilized for evaluating patient outcomes.

## Results

### Patient characteristics

Seventy-seven DRPs underwent exome sequencing following SCT, and were assessed by *in silico* analysis for alloreactivity potential derived from non-synonymous SNPs in the GVHD direction as previously described. [15] The cohort comprised of 27 MRD and 50 MUD SCT recipients underwent data collection for retrospective analysis including GVHD occurrence and hCMV reactivation information S1 Table. Of the 30 patients that experienced hCMV viremia within a median of 29 days from transplant, 26 were hCMV-seropositive or reactivated; one patient had drug-refractory hCMV infection, and succumbed to it. CMV reactivation was experienced in conjunction with GVHD in 18 of the 30 patients, 13 of whom experienced hCMV reactivation prior to GVHD onset. Multiple recurrences of hCMV viremia were experienced in 11 of the 30 patients (2 or more separate reactivation events, ≥200 copies of hCMV DNA/μL plasma per event by quantitative polymerase chain reaction).

### CMV—Human HLA bound peptide homology

Based on the hypothesis that significant amino acid sequence homology between human- and viral-derived peptides presented on the same Class I HLA molecules may lead to donor CD8+ T cell cross reactivity, a bioinformatic pipeline was utilized to assess sequence homology between the patient’s putative alloreactive peptide library, and hCMV-derived oligopeptides bound to each recipient’s HLA. Whole exome sequencing of HLA matched SCT donors and recipients revealed 2,463 ± 603 nsSNPs (mean±SD) with a GVH direction per MRD and 4,287 ± 1154 nsSNPs per MUD recipient (Student’s T-test, p<0.001). Following ANNOVAR 9-mer peptide determination and completion of the sliding window analysis of the resulting 17 amino acid variant oligopeptide resulting from each nsSNP, 43,705 ± 10,938 nonameric potentially alloreactive human peptides were identified per MRD recipient, and 77,025 ± 21,170 per MUD recipient. These were organized into a library by HLA-specific binding affinity (IC50: 0–50,000 nM). Next, each alloreactive Human 9-mer peptide library was further evaluated for their degree of match with the 70686 nonamer peptides derived from the hCMV proteome, utilizing the HLA-specific algorithmic CROSS database. This comparison determined sequence homology for strings of ≥6/6 consecutive amino acids between the two sets of Human and hCMV peptides, and was termed ‘sliding window-match analysis’. This initial screen yielded an average of 29,659 ± 9039 total peptide matches per MRD patient and 52,910 ± 16122 total peptide matches per MUD patient to the hCMV proteome (Student’s T-test, p<0.001) following analysis (S1 Fig). The program output reported for each peptide match, the geneid’s, the HLA, IC50 values (Range: 0–50,000 nM) and complete peptide sequence, as well as the shared partial peptide for degree of sequence homology.

**Fig 3:**
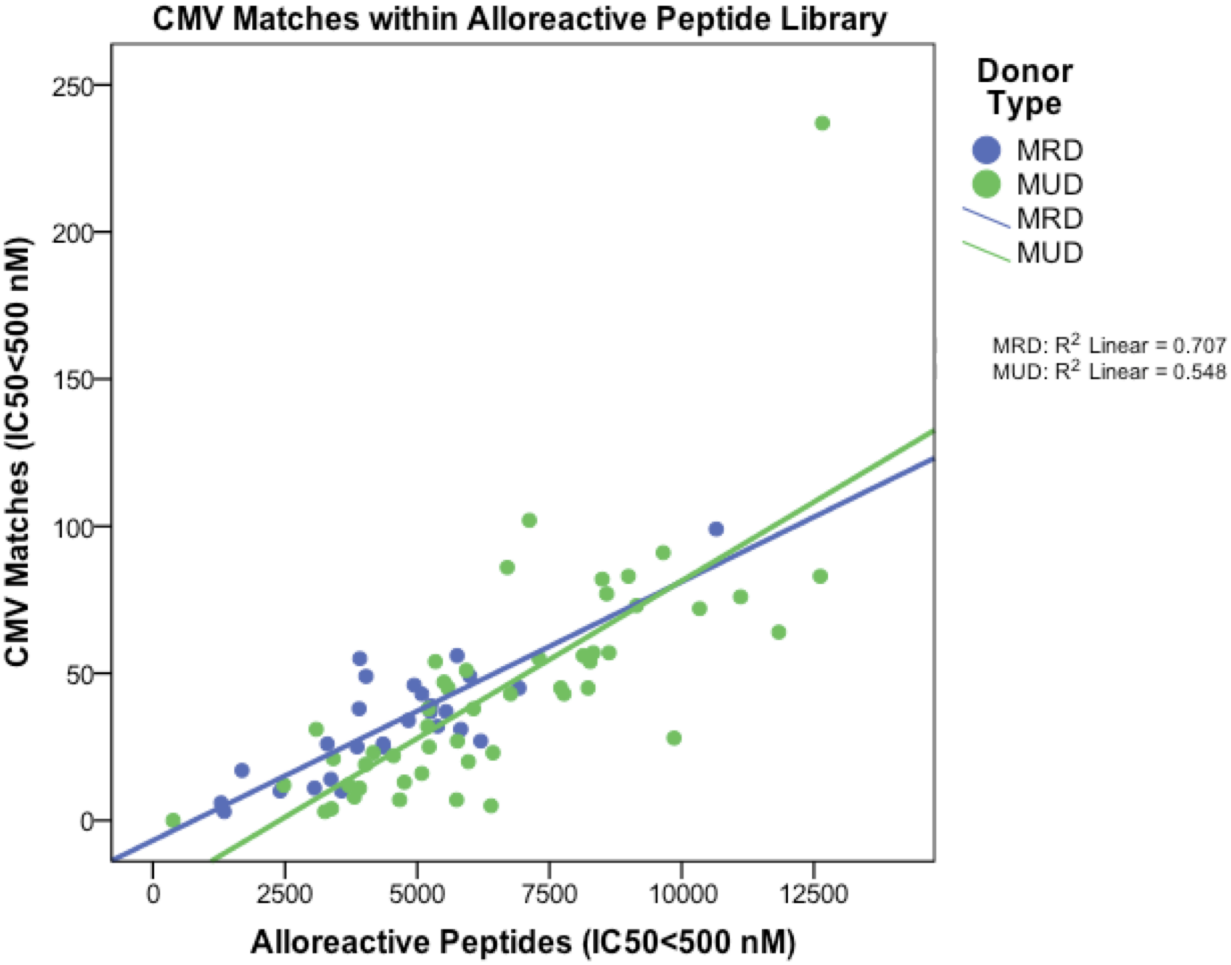
CMV+GVHD cross reactivity potential. Patient-specific peak CMV peptide matches intersecting peak alloreactive peptides (IC50<500 nM) as a cross-reactivity potential stratified by donor type contained within each DRP alloreactive peptide library.

Upon confirmation that all 77 patient alloreactive peptide-HLA libraries studied had matches with the hCMV peptide-HLA arrays, the degree of sequence homology in tightly HLA bound peptides (IC50: 0.01–500 nM) was determined. Following removal of duplicate peptide sequences, the total CMV-human match (homology) library, yielded an average of 33 peptide matches per MRD patient and 44 peptide matches per MUD patient (Student’s T-test, p = 0.09), constituting approximately 1% of the total matches reported (Table 2). Considerable variability was observed in the *in silico* HLA presentation of human-CMV sequence homologous peptides among the different HLA in the DRP studied (S2 Fig). Analysis of the number of Human—CMV matches with IC50<500 nM yielded a lack of association between how common different HLA molecules are within a given population and the overall average or peak number of matches per HLA. The most common HLA-A (02:01) to our patient population was outside the top ten molecules to have the highest average or peak number of Human—CMV matches. The homologous sequence information per patient’s peptide library following high affinity match analysis (IC50<500) was compiled into S2 File; this includes an immunogenicity sorting performed for hCMV reactivating patients. Considering the degree of sequence homology present between hCMV and human peptides bound to HLA class I molecules in specific DRP, each MRD DRP on average had 31, 2, 0 and 0 relevant nonameric CMV-Human peptide matches with 6/6, 7/7, 8/8 and 9/9 sequence homology respectively (Table 2). Also each MUD DRP on average had 40, 3, 1, and 0 peptide matches with 6/6–9/9 sequence homology respectively. As expected from the donor type SNP differences, relevant alloreactive peptide differences, and total peptide matches with hCMV, MUD DRPs exhibited a trend for a higher number of hCMV matched peptides than MRD DRPs by peptide sequence homology differences (6/6-9/9 matching; student’s T-test, p = 0.07–0.38 respectively). To determine the correlation of the number of high affinity CMV-Human matches (IC50<500 nM) with sequence homology >6/6 amino acids in 76/77 patients, these were plotted against the pool of total alloreactive peptides per patient (Fig 3). Notably, MUD DRPs post screening (IC50<500 nM) exhibited a mean of 6,545 ± 2689 alloreactive peptides per patient which was significantly greater than the 4,522 ± 1915 mean alloreactive peptides per MRD DRPs (Student’s T-test, p<0.001) prior to interrogating for matches to the hCMV proteome, with a trend for a higher prevalence of CMV-Human matches in the MUD recipients. These results indicate that there exists a pool of homologous-CMV-derived peptides, which may be presented by the recipient HLA.

**Table 2:** CMV- Human match analysis per DRP (IC50 <500 nM) by donor type according to continuous amino acid sequence homology (one standard deviation from the mean value).

Mean Relevant Matches (IC50<500)	Number of Patients (n)	Sequence Homology 6/6 (±SD)	Sequence Homology 7/7 (±SD)	Sequence Homology 8/8 (±SD)	Sequence Homology 9/9 (±SD)	Total 6/6 to 9/9 (±SD)
**MRD**	27	31 ± 19	2 ± 2	0 ± 1	0 ± 0	33 ± 20
**MUD**	50	40 ± 33	3 ± 6	1 ± 2	0 ± 1	44 ± 39
**Overall**	77	37 ± 29	3 ± 5	1 ± 2	0 ± 1	40 ± 34
**Student's T-test (p < .05)**		0.12	0.09	0.07	0.38	0.09

### Potential immunogenicity of the hCMV peptides presented on recipient HLA

To compare the sequence homology analysis results and determine the presence of previously validated immunogenic hCMV peptides, [36] the specific hCMV peptide sequences and the proteins of origin were reviewed (Table 3). Twelve of the 13 patients with hCMV reactivation before GVHD onset exhibited one or more immunogenic hCMV peptide matches, derived from CMV protein previously shown to elicit a hCMV-specific T-cell response targeting the listed source genes. The remaining patient expressed CMV—Human peptide-matches derived from proteins specific to the patient’s affected GVHD tissue, which had not previously been reported in other studies. The analysis revealed multiple CMV-Human peptide matches that were, immunogenic (IC50<500 nM) and expressed in a GVHD-affected tissue-specific manner. Further, the binding affinity (reflected by the IC50 values) of hCMV peptides when plotted against the IC50 values of the alloreactive Human peptides S3 Fig, demonstrated a wide range of values, indicating the potential for varying degrees of cross reactivity. This analysis illustrates the magnitude of overlap in peptide sequence between the two sources of HLA presented peptides.

**Table 3:** GVHD tissue-specific immunogenic CMV peptide matches, CMV reactivation before GVHD patients. Patient-specific process of cross comparison along with the source genes, IC50 values to indicate inverse binding affinity and GVHD organ specific tissue involvement. *Note: Twelve patients with CMV reactivation/infection before GVHD onset exhibited previously identified immunogenic CMV peptide matches with gene expression specific to the tissues affected by GVHD (above); The filter of immunogenicity provides a connection to T cell reactivity shown *in vitro* to the listed CMV genes in a separate patient population [36]; Patient 79 with muscle/fascia GVHD showed no muscle-specific previously *known immunogenic* CMV peptide matches but still had three relevant CMV peptide matches expressed in the skeletal muscle (not shown); Tissues in parentheses were also affected by GVHD but without immunogenic matches/expression by patient; Patients 67 and 71 experienced *de novo* CMV infection; All 13 patients exhibited multiple CMV peptide matches with unknown immunogenicity.

Patient	HLA	Human Gene	IC50 Value	DRP Peptide	CMV Peptide	IC50 Value	CMV Gene	GVHD Organ with Target Gene Expression
10*	HLA-B40:01	TSPYL1	12	**SETVAL**PPL	QEN**SETVAL**	23	UL86	Skin
	HLA-B40:01	HDAC7	27	EEV**EAVTAL**	AER**EAVTAL**	20	UL49	
	HLA-B08:01	APEH	487	**NRRSAL**YSV	MWK**NRRSAL**	11	UL37	
	HLA-B08:01				WK**NRRSAL**L	274	UL37	
27*	HLA-C03:04	IL22RA1	16	IVH**PTPTPL**	SST**PTPTPL**	42	UL82	Skin
	HLA-A02:01	TECR	28	A**LFSLVV**FT	Y**LFSLVV**LV	3	US3	
	HLA-A02:01				LVY**LFSLVV**	442	US3	
	HLA-C03:04	WNK1	41	I**TAAATA**PV	T**TAAATA**TV	199	UL105	
45*	HLA-A68:02	PPP1R15A	149	**SSAAAAAA**L	T**AAAAAAAA**	58	IRS1,TRS1	Skin & Vagina
	HLA-A68:02				AT**AAAAAAA**	59	IRS1,TRS1	
	HLA-A68:02				D**AAAAAA**PT	286	IRS1	
	HLA-A68:02				SVS**SSAAAA**	376	UL105	
	HLA-A68:02				SA**SAAAAAA**	469	UL105	
	HLA-A68:02				DAT**AAAAAA**	470	IRS1,TRS1	
	HLA-A68:02	MUC21	106	ESS**TTSSGA**	TTY**TTSSGA**	24	UL86	Vagina
	HLA-A68:02				Y**TTSSGA**KI	140	UL86	
	HLA-A68:02	HOXD13	352	**AAAAAAA**GA	T**AAAAAAAA**	58	IRS1,TRS1	
	HLA-A68:02				AT**AAAAAAA**	59	IRS1,TRS1	
	HLA-A68:02				D**AAAAAA**PT	286	IRS1	
	HLA-A68:02				SAS**AAAAAA**	469	UL105	
	HLA-A68:02				DAT**AAAAAA**	470	IRS1,TRS1	
47*	HLA-B07:05	MUC17	52	**TPVSHT**LVA	**TPVSHT**QPL	8	UL150	GI
	HLA-A30:02	CARS2	343	SPA**SLSSLY**	YFD**SLSSLY**	173	UL29,UL28	
48*	HLA-A31:01	PLXND1	200	**LFVFCT**KSR	N**LFVFCT**ER	58	UL34	GI (Skin & Liver)
	HLA-A31:01	PLXND1	307	VA**LFVFCT**K				
67*	HLA-A02:01	FGFR4	20	R**LLLALL**GV	V**LLLALL**LL	209	US29	GI, Lung (Skin)
	HLA-A02:01	FGFR4	165	**LLLALL**GVL				
68*	HLA-A31:01	ALCAM	188	TYT**LTAVRR**	ASL**LTAVRR**	68	IRS1,TRS1	GI, Liver (Skin)
70*	HLA-C07:02	LCN2	122	SY**PGLPSYL**	MR**PGLPSYL**	50	UL75	GI
	HLA-B35:01	SERPINB4	97	E**AAAATA**VL	CA**AAAATA**A	393	UL105	
	HLA-A03:01	FYCO1	488	RAS**LKRLVK**	ST**LKRLVK**K	83	UL69	
71*	HLA-C03:03	PPP1R15A	19	SS**AAAAAA**L	**AAAAAA**PTV	48	IRS1,TRS1	Skin
	HLA-C03:03				**AAAAAAAAA**	343	IRS1,TRS1	
	HLA-C03:03				T**AAAAAAAA**	389	IRS1,TRS1	
	HLA-C03:03				SA**SAAAAAA**	421	UL105	
	HLA-A30:02	CARS2	343	SPA**SLSSLY**	YFD**SLSSLY**	173	UL29,UL28	
76*	HLA-A03:01	ZZEF1	119	**SVLSELL**KK	**SVLSELL**NK	91	UL54	GI
	HLA-B27:05	MUC4	414	T**RHATSL**PV	YQL**RHATSL**	473	US29	
84*	HLA-A02:01	FGFR4	20	R**LLLALL**GV	V**LLLALL**LL	209	US29	GI (Skin)
	HLA-A02:01	FGFR4	165	**LLLALL**GVL				
	HLA-C02:02	MUC17	270	TSM**PVSTTV**	ISY**PVSTTV**	215	UL75	
	HLA-B27:05	MUC4	414	T**RHATSL**PV	YQL**RHATSL**	473	US29	
90*	HLA-A02:01	PELP1	78	**LLALLL**APT	**LLALLL**LEL	38	US29	Skin & GI
	HLA-A02:01				VL**LLALLL**L	209	US29	
	HLA-A02:01	AHSA1	321	KT**LFLAVQ**V	E**LFLAVQ**FV	145	UL86	
	HLA-C03:03	TCF7L1	107	**AAASSS**GQM	**AAASSS**SAV	27	UL48	GI
	HLA-C03:03				I**AAASSS**SA	220	UL48	
	HLA-A03:02	ZZEF1	325	**SVLSELL**KK	**SVLSELL**NK	185	UL54	
	HLA-C03:03	PLEKHA6	385	**AASSSL**RRL	FGG**AASSSL**	71	UL122	

### Tissue expression of cross-reactive human peptides

In order to study the GVHD tissue-specific expression of the hCMV peptides matched to human alloreactive peptides (Table 3), gene expression data organized in a tissue specific manner was obtained from the GTEx Portal of the Broad Institute of MIT and Harvard (Version 6). [37] Gene expression data for all CMV-Human peptide matches in the 30 hCMV reactivating/*de novo* infected patients was compiled (median minimum threshold of expression: ≥10 reads/kilobase of transcript/million mapped reads), specifically focusing on GVHD target tissues including: skin, GI, liver, lung, and others (vagina, muscle, adipose and salivary gland). A focused analysis of GVHD tissue gene expression in conjunction with actual GVHD occurrence was performed on 18 patients with GVHD and hCMV reactivation, and showed that 18/18 patients had expression of hCMV-matched alloreactive peptides (IC50<500 nM) with ≥10 RPKM tissue-specific gene expression (Table 4). As depicted, combining both match data and gene expression data, a cross reactivity profile was created for the hCMV infected subset of patients to include: match number, discrete numbers of peptides (Human and CMV), gene count, immunogenic hCMV genes, and tissue specific GVHD peptides or genes. These data points taken together comprise a predictive case for potential alloreactive trigger following hCMV infection if human and CMV peptide libraries possess enough peptide sequence homology that may be derived from whole exome sequencing of transplant donors and recipients using this analytic approach.

**Table 4:** Human-CMV short sequence homology in GVHD tissue specific peptide and gene distribution from GTEx analysis (n = 18). GVHD incidence denotes the specific organs affected in each patient; Peptides, lists the number of unique peptide-HLA complexes matched between human and CMV peptide library; the column, *Genes* lists the source genes for the aforementioned peptides; *GVHD tissue specific peptides* lists the number of peptides which bind HLA with an IC50 <500nM, and are expressed in tissues affected by GVHD; *GVHD tissue gene expression* denotes the number of genes expressed at an RPKM >10 corresponding to the GVHD tissue specific peptides. Note: *- All patients with an asterisk following their numeric representation experienced CMV reactivation prior to GVHD (except Patients 67 and 71, *de novo* CMV infected) and patients without an asterisk experienced GVHD prior to CMV reactivation. **- human peptides may have overlapping areas of homology yielding a higher number of matches. Immunogenic CMV genes in this context refer to the genes associated with HCMV ORF-specific CD8+ T memory cell responses measured in frequency among CMV seropositive patients previously [36].

Patients	GVHD Incidence						Peptides**			GVHD Tissue Specific Peptides (IC50: <500 nM)						Genes			GVHD Tissue Genes (RPKM: >10)					
n = 18	Skin	GI	Liver	Lung	Vaginal	Muscle	Human	Matches	CMV	Skin	GI	Liver	Lung	Vaginal	Muscle	Human	Immunogenic CMV	CMV	Skin	GI	Liver	Lung	Vaginal	Muscle
10*	**X**						58	72	58	10						47	10	35	8					
27*	**X**						43	64	55	7						39	9	35	7					
45*	**X**				**X**		17	45	26	7				9		15	5	15	6				8	
47*		**X**					21	23	22		6					20	5	19		6				
48*	**X**	**X**	**X**				24	25	21	5	11	5				21	5	18	4	9	4			
67*	**X**	**X**		**X**			28	43	36	3	8		9			24	4	24	3	7		7		
68*	**X**	**X**	**X**				10	10	10	2	7	2				10	2	10	2	7	2			
70*		**X**					25	28	22		9					21	5	21		8				
71*	**X**						29	55	39	2						28	8	30	2					
76*		**X**					10	11	11		5					10	4	10		5				
79*						**X**	39	49	41						3	28	7	26						2
84*	**X**	**X**	**X**				21	25	21	1	7	2				18	4	14	1	6	1			
90*	**X**	**X**					39	56	43	8	17					36	7	27	7	16				
34	**X**						38	57	47	8						32	12	31	7					
38	**X**						32	47	41	6						30	6	29	6					
41		**X**					12	12	11		5					11	2	11		4				
73	**X**	**X**					5	5	5	1	2					5	0	4	1	2				
81		**X**					10	17	10		4					6	0	6		2				

### Clinical correlations of CMV reactivation with GVHD

In this cohort hCMV reactivation following SCT, was associated with poorer survival (n = 46, Log Rank: 4.5, p = 0.03, Hazard ratio: 2.31, p = 0.04) when adjusting for hCMV reactivation by donor or recipient seropositivity S4 Fig, consistent with the findings of a recent CIBMTR analysis. [2] Further we observed a trend of poor survival among CMV seropositive DRPs that experienced hCMV reactivation along with GVHD S5 Fig (n = 46, Log Rank: 3.04, p = 0.08, Hazard ratio: 2.02, p = 0.09). To determine whether there was any association between onset of hCMV reactivation and GVHD, we evaluated lymphocyte growth kinetics and large variations in calcineurin inhibitor levels as possible triggers of GVHD occurrence in the above referenced patients (Table 4). Lymphoid recovery patterns were examined among these patients with early lymphocytosis seen in patients with CMV viremia S6 Fig. GVHD was also examined for association with lymphoid recovery rate and magnitude along with CMV reactivation. CMV reactivation + GVHD patients did have a trend for more frequent occurrence of earlier and larger magnitude lymphoid recovery patterns compared with GVHD alone, CMV alone or the patients with neither. There was however no significant difference in survival between these patient groups S7 Fig. Importantly in the patients with an observed association between CMV reactivation, lymphocytosis and GVHD onset, the T cells were found to be donor-derived DNA by chimerism analysis in most of our patients.

In the 30 hCMV-reactivating patients examined S2 File, reactivation events were often coincident with GVHD onset and associated with preceding lymphocytosis, suggesting an alloreactive cellular immune response S8 Fig (Panels A, B and C). Acute GVHD onset following hCMV reactivation was seen in 7 of the 13 patients experiencing hCMV reactivation before GVHD; 3 of these patients developed grade IV gastrointestinal GVHD (2 steroid refractory) which was fatal in all cases. As seen in figure S8 Fig (Panel A), with acute GVHD in patients 47 and 68, there was evidence of hCMV reactivation events (top graph) that preceded rapid onset lymphocytosis (middle graph) and often occurred with stable CNI or immunosuppression levels (bottom graph), indicating a potential hCMV-GVHD relationship. Patients 47 and 68 with acute GI GVHD had an average of 16.5 ± 9.2 matches (±SD) derived from 15.5 ± 7.8 human peptides. Patient 68 also exhibited skin and liver GVHD. When considering the percentage of genes with GVHD-tissue specific expression on average, in these two patients 87.5 ± 17.7% peptides were derived from GI tract specific alloreactive-hCMV matched genes (primary GVHD organ).

Patients depicted in S8 Fig (Panel B, patients 10 and 27) exhibited the more stable or gradual hCMV reactivation effects seen in chronic GVHD, where lower or less frequent elevations in hCMV titers may still elicit lymphocyte growth, albeit at a slower rate, probably accounting for the observed difference in outcomes between patients with symptoms of only acute GVHD or chronic GVHD. Patients 10 and 27 with Chronic Skin GVHD had a mean of 68 ± 5.7 matches (±SD) derived from 50.5 ± 10.6 human peptides. These two patients exhibited a lower percentage of GVHD-tissue specific gene expression on average when considering all potential GVHD genes identified, in this instance skin gene expression amounted to 51.9 ± 2.7%.

The final S8 Fig group (Panel C, patients 71 and 84) exhibited symptoms of both acute and chronic GVHD at different times post transplant with differing disease patterns according to the GVHD tissues involved (i.e., acute skin or GI vs chronic skin or GI GVHD may be defined/graded differently), but patient 84’s lower grade cyclical reactivation events altered the lymphocyte growth pattern towards a recurring waveform pattern, with peaks and troughs of lymphocytes over time. Patients 71 and 84 exhibiting both acute and chronic GVHD had a mean of 40 ± 21.2 matches (±SD) derived from 25 ± 5.7 human peptides. Patient 84 also exhibited GI and liver GVHD. Patients 71 and 84 also exhibited lower GVHD-tissue specific gene expression on average when considering all potential GVHD genes identified, Skin,or GI (primary organs): 44.3 ± 27.3%. There were 3 patients who developed recipient-derived T cell chimerism following CMV reactivation and lymphocytosis (patients 2, 7 and 28), suggesting expansion of recipient derived T cell clones in response to CMV reactivation. These three patients were all CMV sero positive with CMV + donors. These variable dynamics of hCMV reactivation and GVHD onset demonstrate the complexity involved in analyzing the relationship between ongoing immunosuppression in the setting of multiple sets of potentially cross reactive antigens by affected organ system being presented to a reconstituting donor-derived immune system.

## Discussion

CMV reactivation is a frequent complication of allografting, requiring frequent monitoring and associated with an increased risk of treatment related mortality, primarily in its own right, but also because it is frequently associated with GVHD. [12] Therapy and effective prophylaxis involve the use of toxic drugs and monitoring for reactivation is not straightforward. The ability to identify patients at risk of developing alloreactive complications from hCMV reactivation will therefore be a useful adjunct to the supportive care of transplant recipients, as well as an important step forward in understanding virus-induced-alloreactivity. In this paper a computational algorithm that identifies hCMV peptides homologous to human alloreactive peptides is described. This determination required three steps, whole exome sequencing of transplant donors and recipients, followed by *in silico* determination of the patient specific class I HLA binding of the oligopeptides resulting from the nsSNP in the exome and finally a comparison of these alloreactive peptide sequences with those of hCMV peptides predicted to bind the same HLA molecules. This algorithm identifies a number of hCMV peptides which bind the same HLA as human peptides with a similar range of binding affinities and also demonstrate a degree of sequence homology with the human peptides. We hypothesize that these peptides may potentially be cross-presented to donor T cells.

To understand how this may impact GVHD pathophysiology, consider a T cell clone (TC_CMV_), which recognizes an hCMV peptide-HLA complex, is activated by hCMV viremia. The T cell receptor of this clone may also recognize a human alloreactive peptide with sequence homology to the hCMV peptide and bound to the same HLA molecule. Even if it does so weakly, tissue damage may be initiated and GVHD ensue. This process can work in reverse as well, a T cell clone with high affinity for alloreactive peptides (TC_mHA_), which only binds the hCMV peptide-HLA complex weakly may be ‘set-off’ by a hCMV reactivation event, again leading to down-stream GVHD. This general principle may hold true for other virus derived peptides.

A mathematical model utilizing matrices has been developed to understand aggregate T cell responses to many mHA-HLA complexes the donor T cells may encounter in the recipient milieu. [39] The simplifying notion underlying this model is that each donor T cell interacts with a single recipient HLA-bound antigen, therefore an identity matrix may be used to calculate the resulting T cell response. This model requires that the antigens occupy the matrix operator (*M*_*APO*_) and T cell vector gets transformed by the operator (Table 5).

**Table 5:** Matrix depicting the T cell clonal interaction with mHA-HLA.

$M_{APO}$			
	mHA_1_ HLA	mHA_2_ HLA	mHA_n_ HLA
*TC*_*1*_	1	0	0
*TC*_*2*_	0	1	0
*TC*_*n*_	0	0	1

In the above simplified matrix T cell clone (Table 5), TC_1_ interacts with mHA_1_, and so on. The 1 in the cells means that the T cells recognize that antigen and responds, and 0 means absence of recognition, this can also be understood as probability of TCR binding to the peptide-HLA complex. In reality, the TCR-Ag-HLA interactions are not likely to be quite so simple. An important clue to this is the observation that antigen-HLA binding affinities, reported in this paper as IC50, do not take on discrete values of 0 or 1 (binding or no binding); instead there is a continuum of IC50 values. So in the case of cross-reactive antigens, a hCMV derived antigen binds the same HLA molecule and may interact with the T cell receptor, albeit with a different binding affinity. This implies that each TCR might interact with multiple antigens with different affinity, thus the 0 in the matrix above may in reality be replaced with a series of numbers between 0 and 1. These cross-reactive antigens may augment the T cell response to the primary target antigens, as shown in the matrix below where cross reactivity is depicted (Table 6).

**Table 6:** Matrix depicting T cell clonal cross-reactivity between CMVp-HLA and mHA-HLA. *-indicates response of the alloreactive T cell clone to a viral pathogen peptide,bound to the same HLA as the mHA and vice versa. For example, TC_1_ recognizes, mHA_1_ HLA + CMVp_2_HLA, TC_1CMV_ recognizes, mHA_1_ HLA + CMVp_1_ HLA, and so on.

$M_{APO}$				
	mHA_1_-HLA	(CMVp_1_-HLA)	mHA_2_-HLA	(CMVp_2_-HLA)
*TC*_*1*_	1	0.2*	0	0
*TC*_*2*_	0	0	1	0.3
*TC*_*1CMV*_	0.3*	1	0	0
*TC*_*2CMV*_	0	0	0.1*	1

The notion of T cell receptor (TCR) cross reactivity presented here also merits further discussion. An argument may be made that mHA that do not share an entire sequence are unlikely to elicit a response from relevant TCR. However this argument assumes a rigid interaction between the TCR and the mHA-HLA complex. In reality these interactions are likely to be elastic in nature, as can be inferred from the continuum of values that the CMV peptide/mHA-HLA IC50s demonstrate. In a rigid frame work one will likely observe discrete binding affinity value sets. Such ‘elasticity’ in TCR recognition has previously been demonstrated in the context of HLA-B35:01 bound HIV-1 derived Nef epitope VY8. In this study, patient derived CD8+ T cell clones recognized index peptides despite substitution in the AA residues along the peptides, demonstrating unique cross reactivity ‘footprints’ for individual T cell clones. [40,41] The counter argument of multiple T cell clones recognizing a single antigen has also been studied in the context of tumor antigen HLA-A:0201/NY-ESO-1 specific CD8+ T cell clones, where multiple T cell clones with relatively restricted TRB V and J segment usage were identified. These T cell clones were equally efficient and dependent on recognition of the central peptide residues for activity. [42] Similar findings have been reported for melanoma tumor infiltrating CD8+ T cells which recognize variants of HLA-A2 bound MART-1 antigen variants. [43] Other models of T cell cross reactivity have also been reported in the literature for TCR recognition, such as, widely degenerate recognition of unrelated peptides in peptide-MHC complexes exhibiting docking geometry diversity and CDR Loop displacement. [16,44–46] Alternatively, TCR recognition of only closely related peptides to original pathogen-derived peptides with the same docking geometry of binding and similar CDR3 displacement patterns has been proposed more recently. [47] Another consideration is the differential effect of binding affinity and stability of CMV peptide/mHA—HLA interactions influenced by enhanced antigen availability by either greater proteolytic activity [48] or related to higher tissue expression levels. [49–51] Taken together these lines of evidence suggest that TCR cross reactivity, and stimulation of alloreactivity is certainly possible in the context of the same HLA molecules presenting both CMV and alloreactive human peptides with sequence homology. It is however important to recognize that this mechanism most likely only contributes to the initiation of GVHD in some patients, with more conventional alloreactive T cell responses occurring in the face of tissue injury and systemic inflammation being responsible for GVHD in most cases. [52] While the *in silico* analysis for hCMV + alloreactivity potential reveals a large body of antigens which may influence clinical outcomes. However, there are caveats to be considered in developing this peptide analysis pipeline. First, the process of creating the CMV CROSS database included some variant forms of the hCMV proteins known to have differing amino acid lengths that may allow for duplicates, which were accounted for during multiple processes of deduplication. However the slight differences to the variant forms of the proteins reported in the NCBI database may generate a few more peptide possibilities than may be realistic but would allow for strain differences of the human peptide matches during exome sequencing.

An interesting observation among alloreactive or hCMV peptides was where human genes and hCMV immunogenic genes were shared among multiple patients with many of each gene or peptide involved being common in our cohort, often with the same HLA specificity and predicted binding affinity S2 File. Epitope spreading is another phenomenon, that may be exemplified by donor lymphocytes and antigen presenting cells encountering either hCMV or recipient alloreactive peptides released upon organ damage that may be processed and presented in an HLA restricted manner and elicit an immune response. CMV has evolved in parallel with the human genome over hundreds of millions of years, potentially exchanging genetic information from virus and human randomly with each latent infection, selecting primarily for immune evasion. [53] This phenomenon shields hCMV from immune response but also exposes endogenous antigens from recipient cells simultaneously with hCMV antigens to donor immune surveillance during organ damage from lytic reactivation in athymic adults. [54] This form of epitope spreading, developing T cell antigenic experience, affects overall immune responses and may account for the robust CD8+ T cell response to hCMV infection in otherwise healthy individuals (10% of the entire T cell compartment) that inflates with age. [36] In addition, the primarily memory T cell response to hCMV accounts for the sharp responses to various hCMV antigens upon reactivation, which may be as great as 50 fold the strength of a naïve T cell response during primary infection. [19] Recently, reduced TCR repertoire diversity in naïve T cells has been reported in patients who have CMV reactivation and GVHD following MUD SCT. The authors evaluated repertoire deficiencies by comparing patient samples with reference and found that patients who had CMV reactivation, with or without GVHD had greater ‘holes’ in the repertoire, suggesting that an oligoclonal population of cross reactive T cells clones may lead to immune dysregulation. [55] Strong CD8+ and CD4+ response to soluble recombinant hCMV antigen has been demonstrated in the past. [56] This may be related to epitope spreading in post-transplant viral immunity, as has already been discovered in Multiple Sclerosis and in other autoimmune disorders. [24,57,58] Nevertheless, GVHD is a condition that is primarily dependent on the presence of an adequate alloreactive stimulus, the abrogation of which would mitigate GVHD regardless of CMV reactivation. This may be observed in recipients of T cell depleted transplants, including some of the patients reported here. Further, when considering the opportunity for hCMV to catalytically trigger GVHD apart from the potential method we propose here, we must note that hCMV is the largest of the herpesvirus family with a 235 kb DNA genome capable of encoding more than 200 potential protein products [59] and second the near ubiquity of cells it is capable of infecting in man, including parenchymal and connective tissue cells of virtually any organ along with various hematopoietic cell types. [60]

In conclusion, this paper reports sequence homology in HLA bound peptide antigens of hCMV and human origin. Given the distribution of human peptides in various tissues, and their involvement with GVHD in the patients examined, we posit that hCMV derived peptides may influence the development of GVHD in patients who develop hCMV reactivation following SCT These findings support the use of more aggressive antiviral strategies for preventing hCMV reactivation in patients undergoing allografting and argue against the use of simply monitoring as the major therapeutic strategy. We endeavor in the future to understand the supporting CD4+ T cell hCMV protective dynamics involved by interrogating HLA class II peptides, (1) looking at potential influences of human CMV mimicry and unraveling the potential GVHD/CMV cross-reactivity/Auto-immune relationship further. [13,18,24,61–65]

## Supporting information

S1 File Appendix A. CMV CROSS database tools excel spreadsheet.
Sheet 1. CMV proteome—NCBI accessions: hCMV/HHV5Sheet 2. CMV immunogenic protein—CD8+ list from Ref. #36.
(XLSX)

S2 File Appendix B. Match analysis excel spreadsheet.
Sheet 1. Raw CMV match algorithm results—pre-IC50 screeningSheet 2. Match analysis summary—total peptide # by patientSheet 3. Match analysis peptides—screened, sorted pt. peptidesSheet 4. All CMV case studies, tables, and figuresSheet 5. Peptide-HLA binding patterns sorted by CMV+GVHSheet 6. Exome dataset—retrospective, de-identified info.
(XLSX)

S1 Fig MUD vs MRD total human—CMV matches. Total number of Human—CMV sequence homologous peptides without binding affinity filtering by IC50 values for TCR relevance. Range of IC50 values: 0.01–50000 nM. Averaged number of total matches for MRD DRP (Left, Blue) versus MUD DRP (Right, Green) were displayed above the graph to indicate the observed difference of CMV peptide matches to alloreactive peptides prior to screening by donor type.
(TIFF)

S2 Fig Match analysis by HLA locus. Average number of HLA bound human—CMV sequence homologous peptides ordered by number of peptides presented by HLA locus. Descending order of average matches per HLA (>5 matches per HLA as threshold). Error bars indicated where HLA molecules were shared by more than one DRP and matches averaged.
(TIF)

S3 Fig Human vs CMV binding affinity patterning to DRP-shared HLA. Binding affinity differences indicated by inverse IC50 values from matched human and CMV peptides bound to the same HLA. Each data point represents the intersection of a matched peptide bound to an HLA class I molecule (CMV→Human). These peptides may be cross reactive, with varying degrees of T cell cross reactivity potential for alloreactivity trigger to ensue (towards the origin on the human peptide axis being the greatest).
(TIF)

S4 Fig Survival by CMV incidence accounting for reactivation. GVHD-independent effects of CMV post SCT in CMV seropositive DRP (n = 46).
(TIF)

S5 Fig Survival by CMV & GVHD incidence accounting for reactivation. Cumulative effects of CMV viremia + GVHD post SCT in CMV-seropositive DRP (n = 46).
(TIF)

S6 Fig Lymphoid recovery pattern by CMV+GVHD status. (Top) Lymphoid Recovery Patterns by ALC value achieved by Day 45 post SCT. Pattern A showed early lymphoid recovery (Patient 2), Pattern B showed a medium time to lymphoid recovery (Patient 48) and Pattern C showed late or very low lymphoid recovery (Patient 16). Trendlines indicated the fit to the logistic pattern previously observed. (Bottom) Lymphoid Recovery Patterns by CMV+GVHD Status (n = 77): CMV Reactivation triggers Lymphocytosis that may influence GVHD onset. Fisher’s Exact Chi Square compared CMV + GVHD groups with lymphoid recovery patterns as shown.
(TIF)

S7 Fig Cumulative survival by CMV & GVHD incidence. CMV reactivation with or without GVHD onset affects the survival negatively compared to the protective GVHD effect.
(TIFF)

S8 Fig GVHD patient case studies with coincident lymphocytosis and prior CMV reactivation, split by onset/ progression type. (A) CMV-reactivation course with acute GVHD onset and progression: Patients 47 and 68. Patient 47, grade IV GVHD of the GI tract; patient 68,steroid refractory grade IV GI tract GVHD, skin and liver. Both patients showed signs of CMV reactivation and bursts of lymphocytosis prior to GVHD onset during stable immunosuppression (TAC, Tacrolimus; SIR, Sirolimus; Cyclosporin) as measured by serum levels or following taper. (B) CMV-reactivation with chronic GVHD onset: Patients 10 and 27. Patient 10 had CMV reactivation prior to a gradual lymphocyte proliferation during stable immunosuppression levels (TAC, Tacrolimus) and eventually developed relapsed malignancy. Patient 27 exhibited a CMV reactivation event prior to lymphocytosis at stable tacrolimus levels, and had mainly skin and oral GVHD. (C) CMV reactivation/de novo infection in patients with aGVHD + cGVHD: Patients 71 (new, continuous infection for 200+ days) and 84 (reactivation). Patient 71 showed poor lymphoid recovery, continuous CMV viremia and eventual relapsed malignancy, also developed aGVHD of the skin grade I and also developed mild cGVHD of skin. Patient 84 exhibited lymphocytosis following CMV reactivation events and eventually developed acute recurrent gut and skin grade IV GVHD. This patient also developed moderate cGVHD of gut, skin and liver. Both patients received tacrolimus.
(TIF)

S1 Table Patient characteristics. (TIF)
