# Supplementary figures and images for: Sequence homology between HLA-bound cytomegalovirus and human peptides: A potential trigger for alloreactivity
Source: PLoS One. 2017 Aug 11;12(8):e0178763. doi: 10.1371/journal.pone.0178763 (PMC5553991; doi:10.1371/journal.pone.0178763)

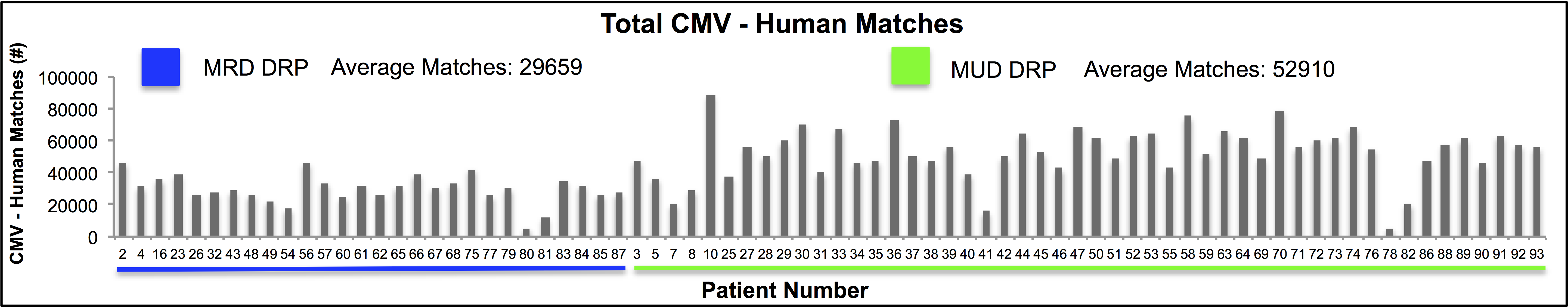

Supplement: S1 Fig — Total number of Human—CMV sequence homologous peptides without binding affinity filtering by IC50 values for TCR relevance. Range of IC50 values: 0.01–50000 nM. Averaged number of total matches for MRD DRP (Left, Blue) versus MUD DRP (Right, Green) were displayed above the graph to indicate the observed difference of CMV peptide matches to alloreactive peptides prior to screening by donor type. (TIFF) [file pone.0178763.s003.tiff]

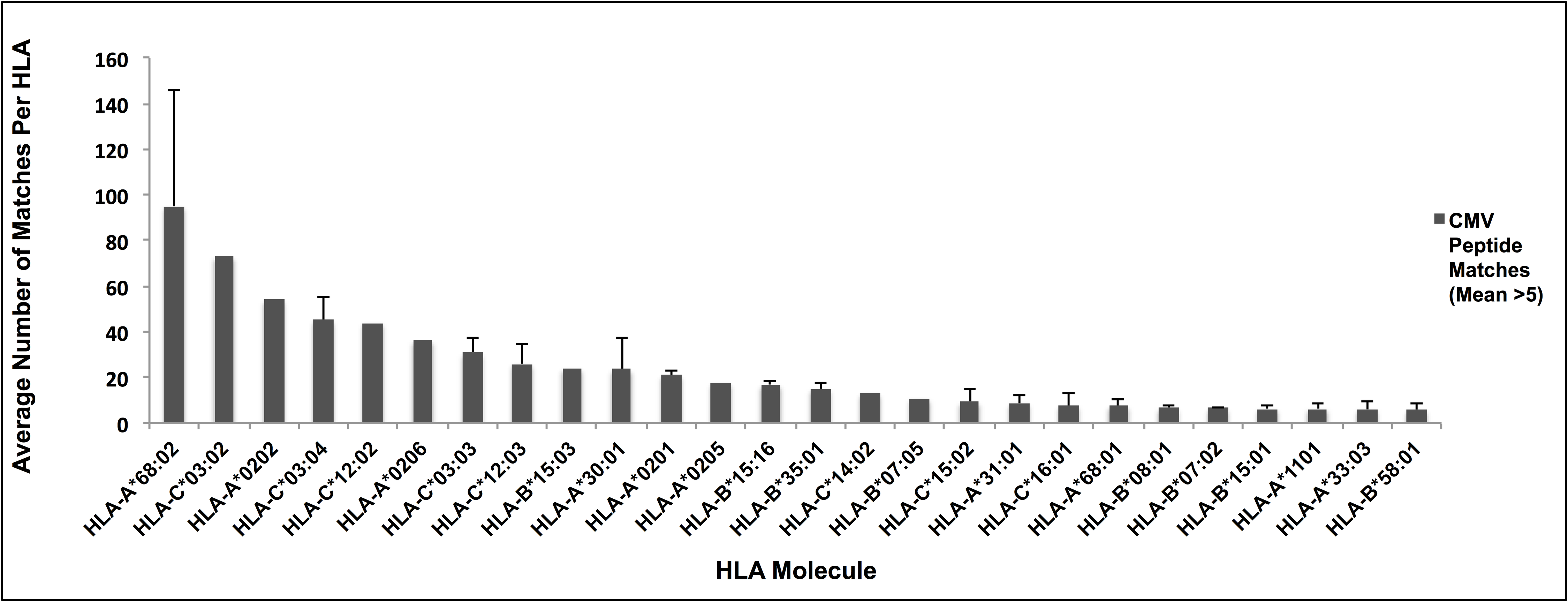

Supplement: S2 Fig — Average number of HLA bound human—CMV sequence homologous peptides ordered by number of peptides presented by HLA locus. Descending order of average matches per HLA (>5 matches per HLA as threshold). Error bars indicated where HLA molecules were shared by more than one DRP and matches averaged. (TIF) [file pone.0178763.s004.tif]

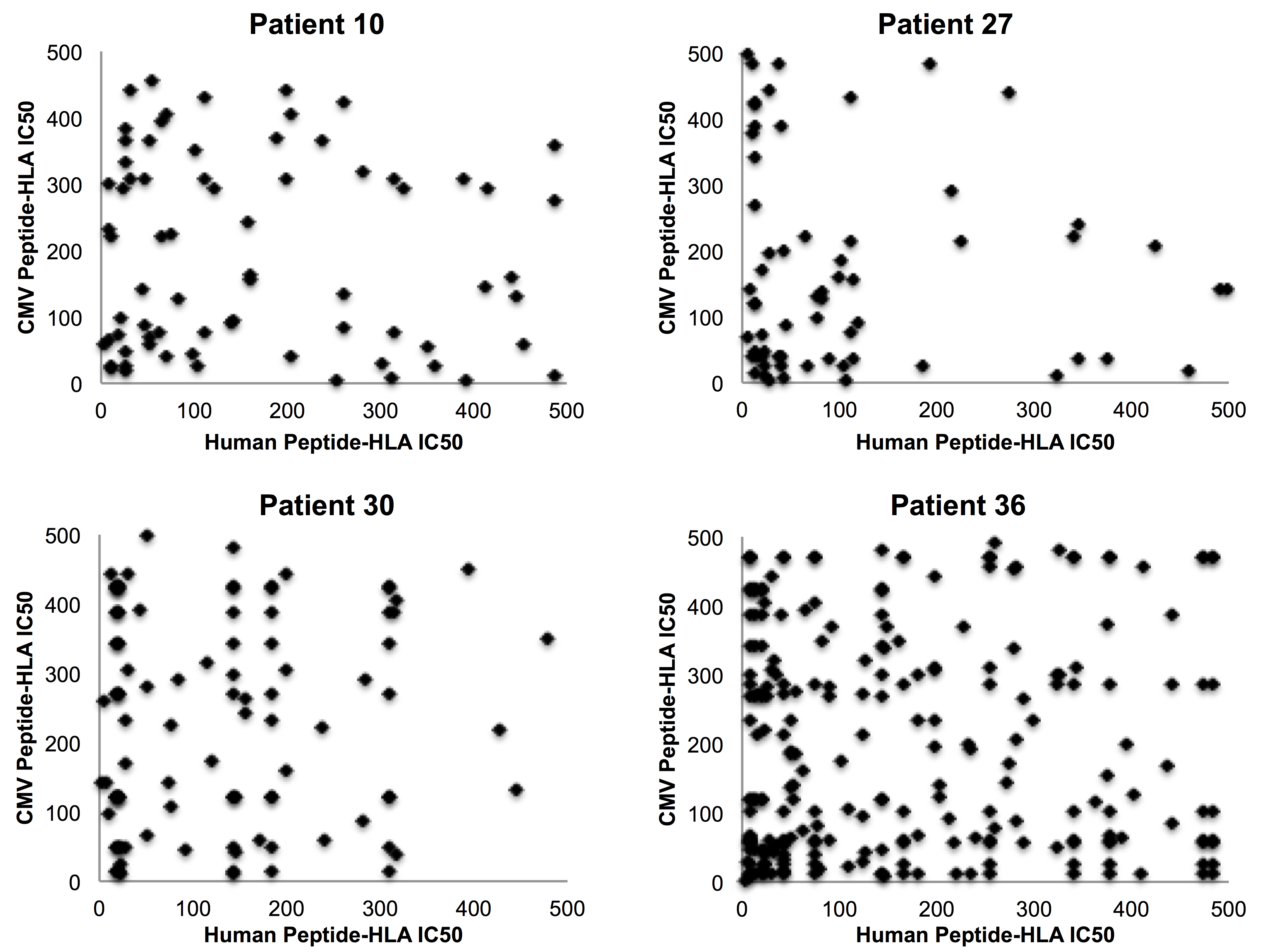

Supplement: S3 Fig — Binding affinity differences indicated by inverse IC50 values from matched human and CMV peptides bound to the same HLA. Each data point represents the intersection of a matched peptide bound to an HLA class I molecule (CMV→Human). These peptides may be cross reactive, with varying degrees of T cell cross reactivity potential for alloreactivity trigger to ensue (towards the origin on the human peptide axis being the greatest). (TIF) [file pone.0178763.s005.tif]

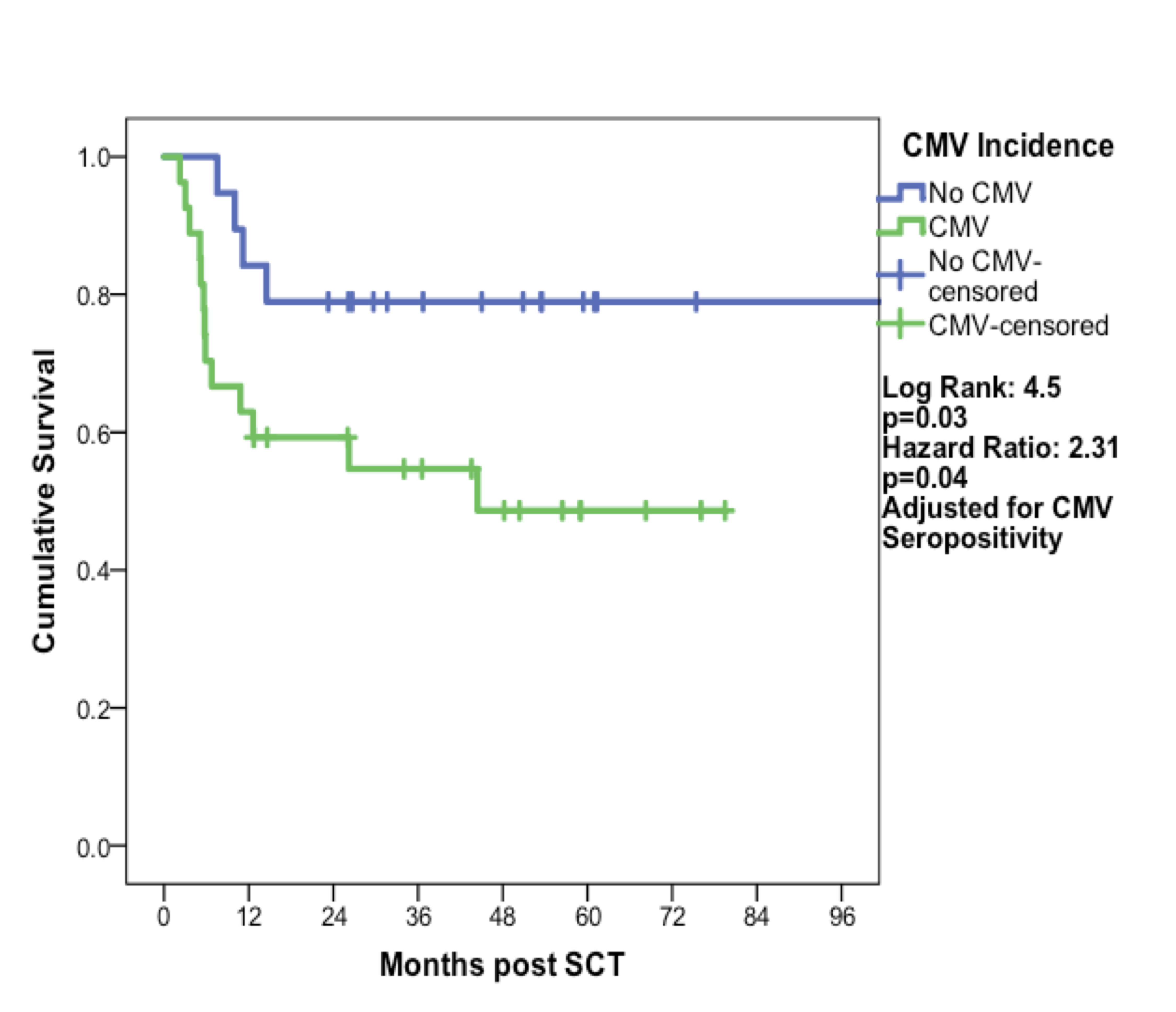

Supplement: S4 Fig — GVHD-independent effects of CMV post SCT in CMV seropositive DRP (n = 46). (TIF) [file pone.0178763.s006.tif]

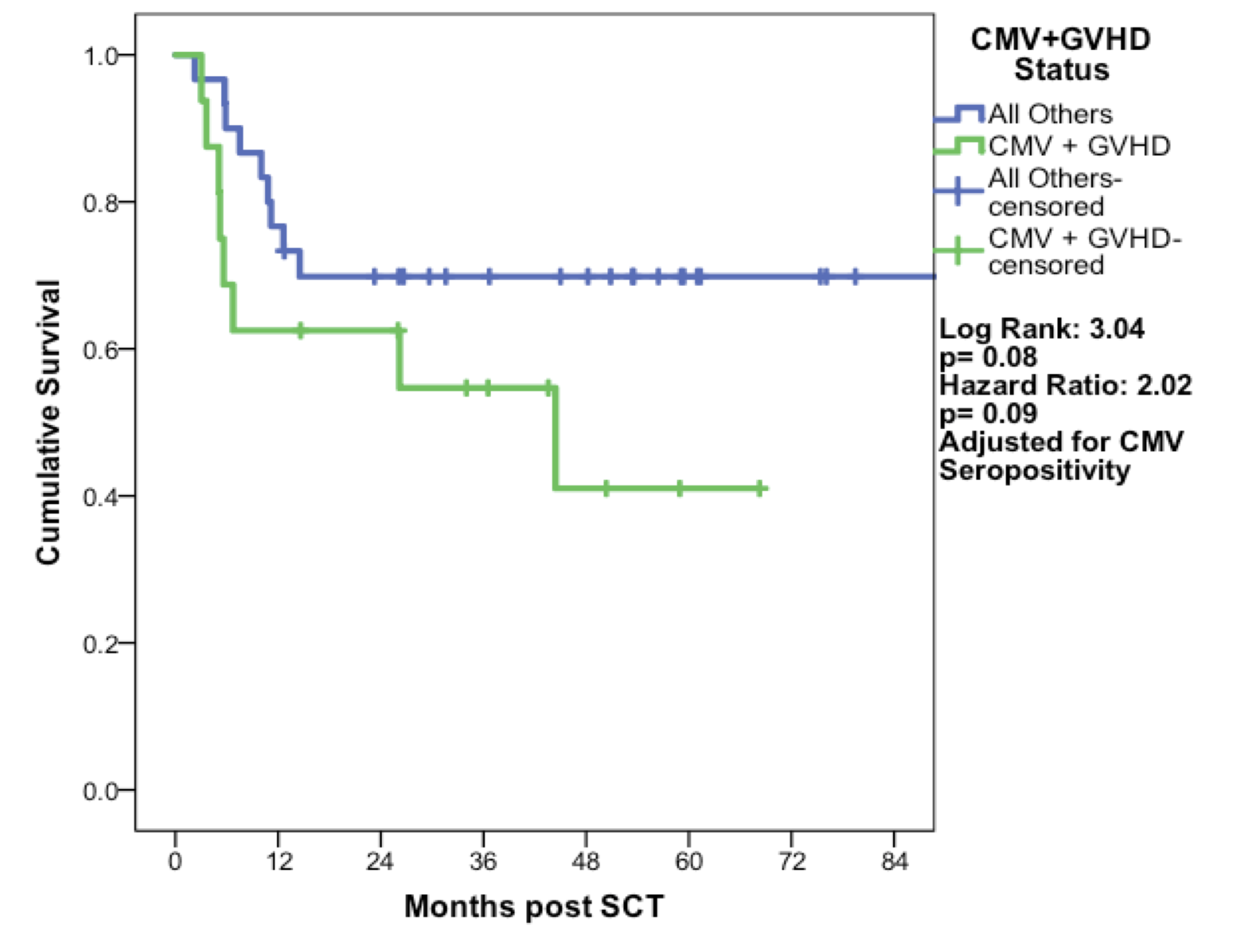

Supplement: S5 Fig — Cumulative effects of CMV viremia + GVHD post SCT in CMV-seropositive DRP (n = 46). (TIF) [file pone.0178763.s007.tif]

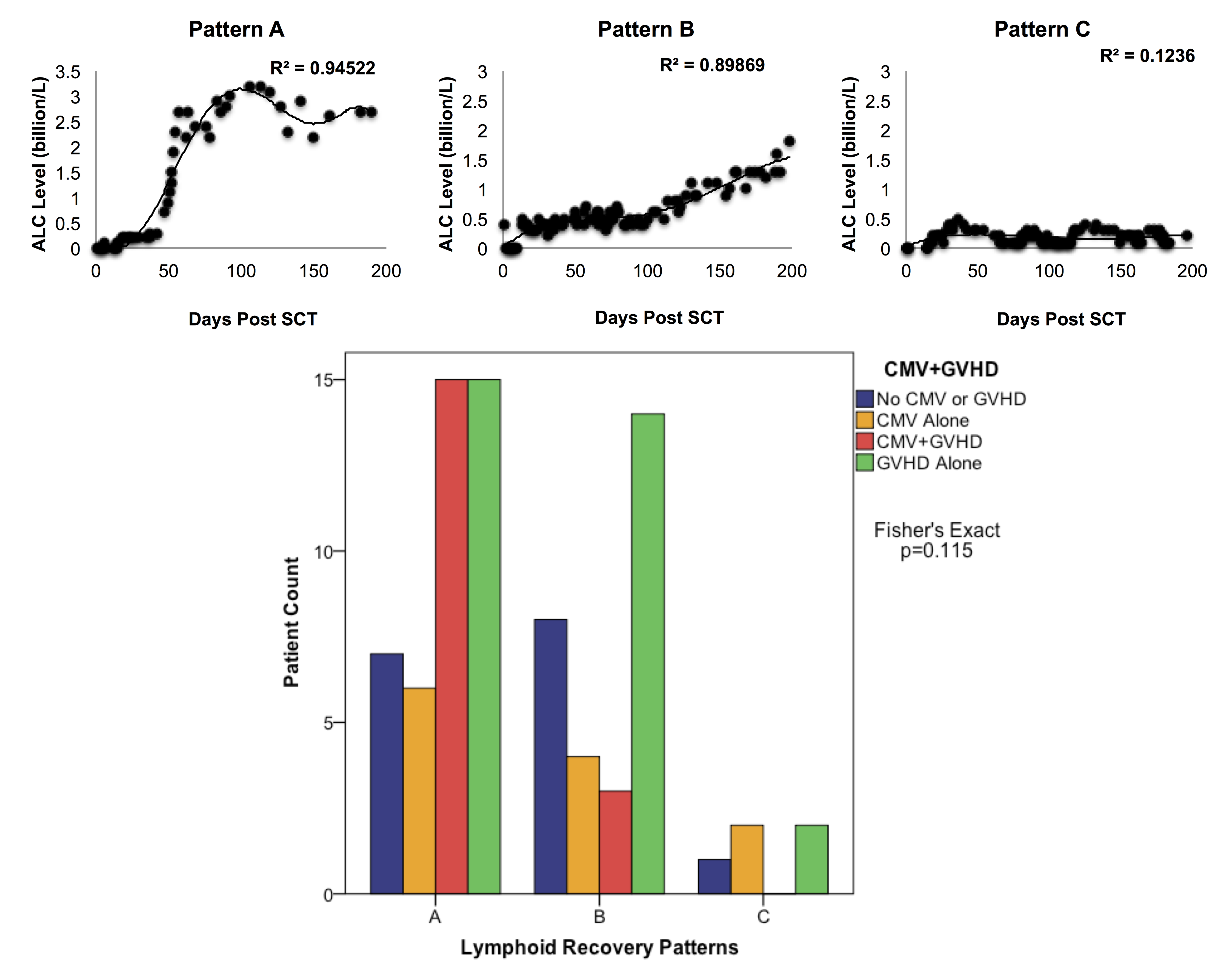

Supplement: S6 Fig — (Top) Lymphoid Recovery Patterns by ALC value achieved by Day 45 post SCT. Pattern A showed early lymphoid recovery (Patient 2), Pattern B showed a medium time to lymphoid recovery (Patient 48) and Pattern C showed late or very low lymphoid recovery (Patient 16). Trendlines indicated the fit to the logistic pattern previously observed. (Bottom) Lymphoid Recovery Patterns by CMV+GVHD Status (n = 77): CMV Reactivation triggers Lymphocytosis that may influence GVHD onset. Fisher’s Exact Chi Square compared CMV + GVHD groups with lymphoid recovery patterns as shown. (TIF) [file pone.0178763.s008.tif]

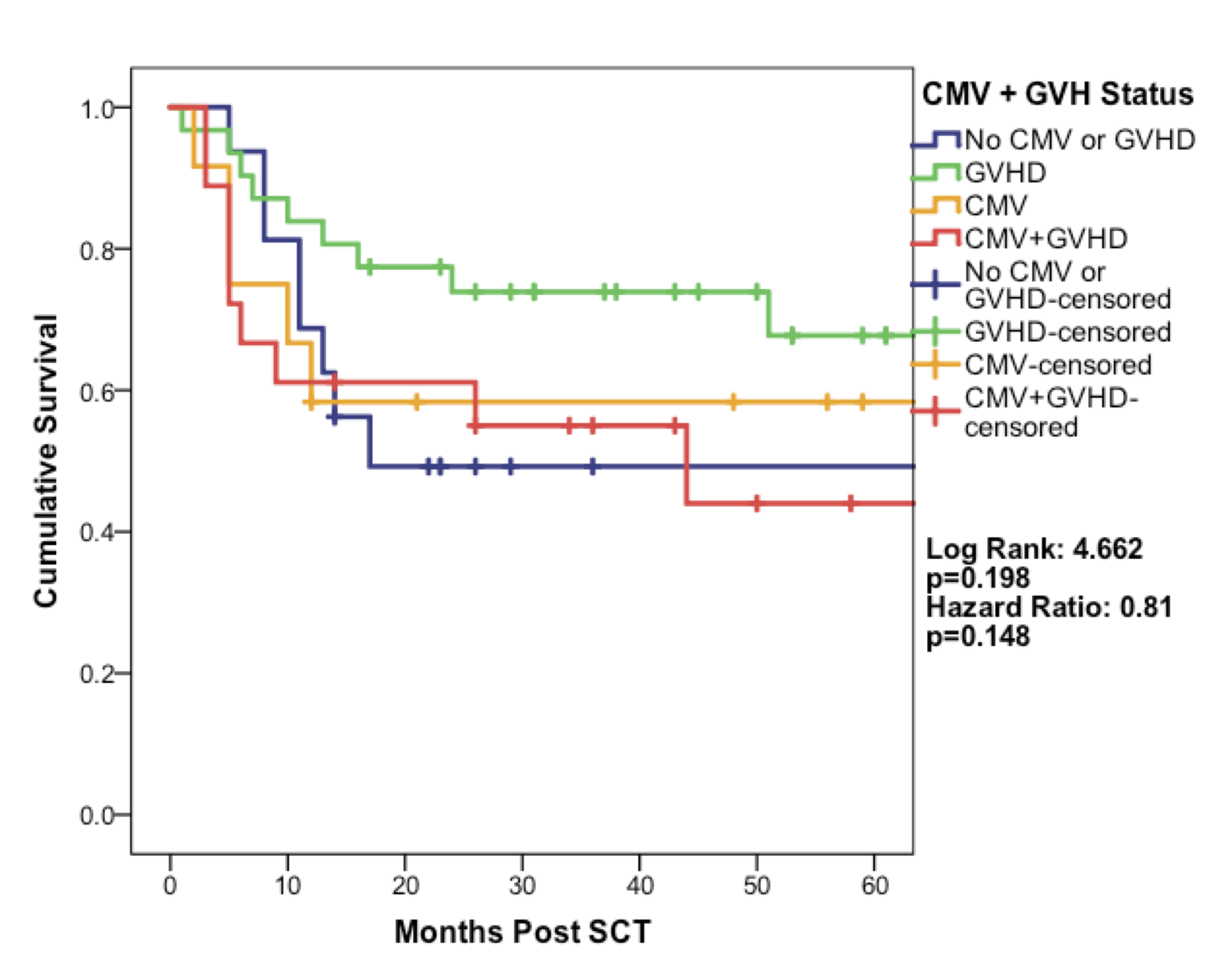

Supplement: S7 Fig — CMV reactivation with or without GVHD onset affects the survival negatively compared to the protective GVHD effect. (TIFF) [file pone.0178763.s009.tiff]

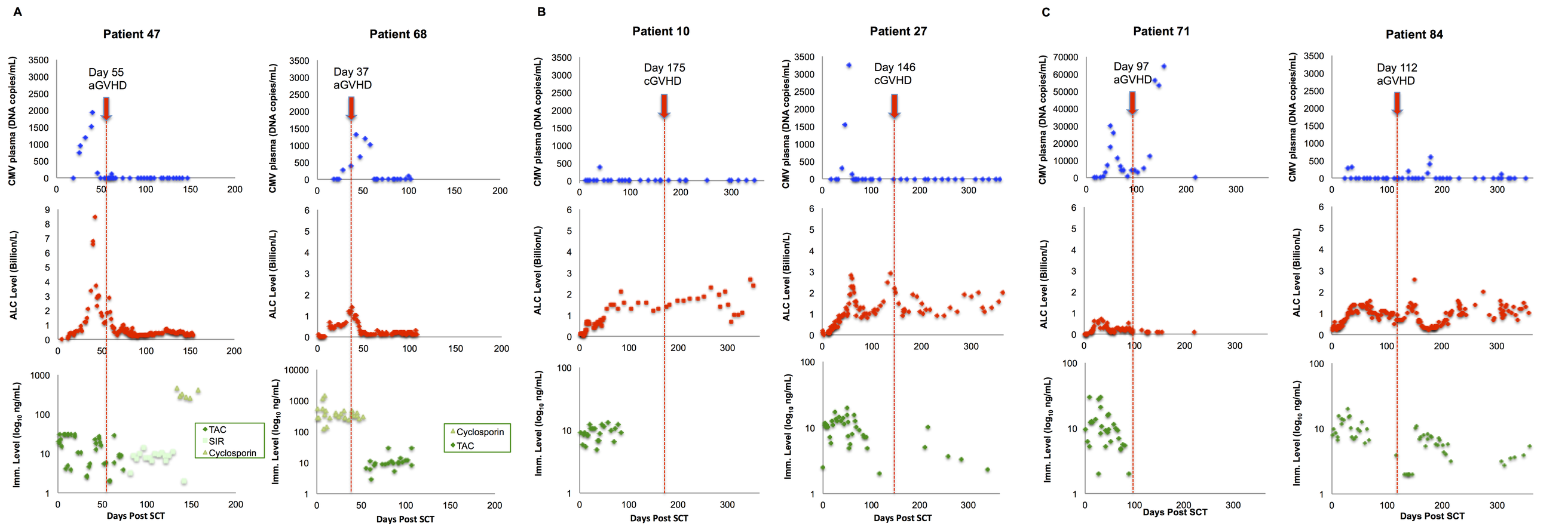

Supplement: S8 Fig — (A) CMV-reactivation course with acute GVHD onset and progression: Patients 47 and 68. Patient 47, grade IV GVHD of the GI tract; patient 68,steroid refractory grade IV GI tract GVHD, skin and liver. Both patients showed signs of CMV reactivation and bursts of lymphocytosis prior to GVHD onset during stable immunosuppression (TAC, Tacrolimus; SIR, Sirolimus; Cyclosporin) as measured by serum levels or following taper. (B) CMV-reactivation with chronic GVHD onset: Patients 10 and 27. Patient 10 had CMV reactivation prior to a gradual lymphocyte proliferation during stable immunosuppression levels (TAC, Tacrolimus) and eventually developed relapsed malignancy. Patient 27 exhibited a CMV reactivation event prior to lymphocytosis at stable tacrolimus levels, and had mainly skin and oral GVHD. (C) CMV reactivation/de novo infection in patients with aGVHD + cGVHD: Patients 71 (new, continuous infection for 200+ days) and 84 (reactivation). Patient 71 showed poor lymphoid recovery, continuous CMV viremia and eventual relapsed malignancy, also developed aGVHD of the skin grade I and also developed mild cGVHD of skin. Patient 84 exhibited lymphocytosis following CMV reactivation events and eventually developed acute recurrent gut and skin grade IV GVHD. This patient also developed moderate cGVHD of gut, skin and liver. Both patients received tacrolimus. (TIF) [file pone.0178763.s010.tif]

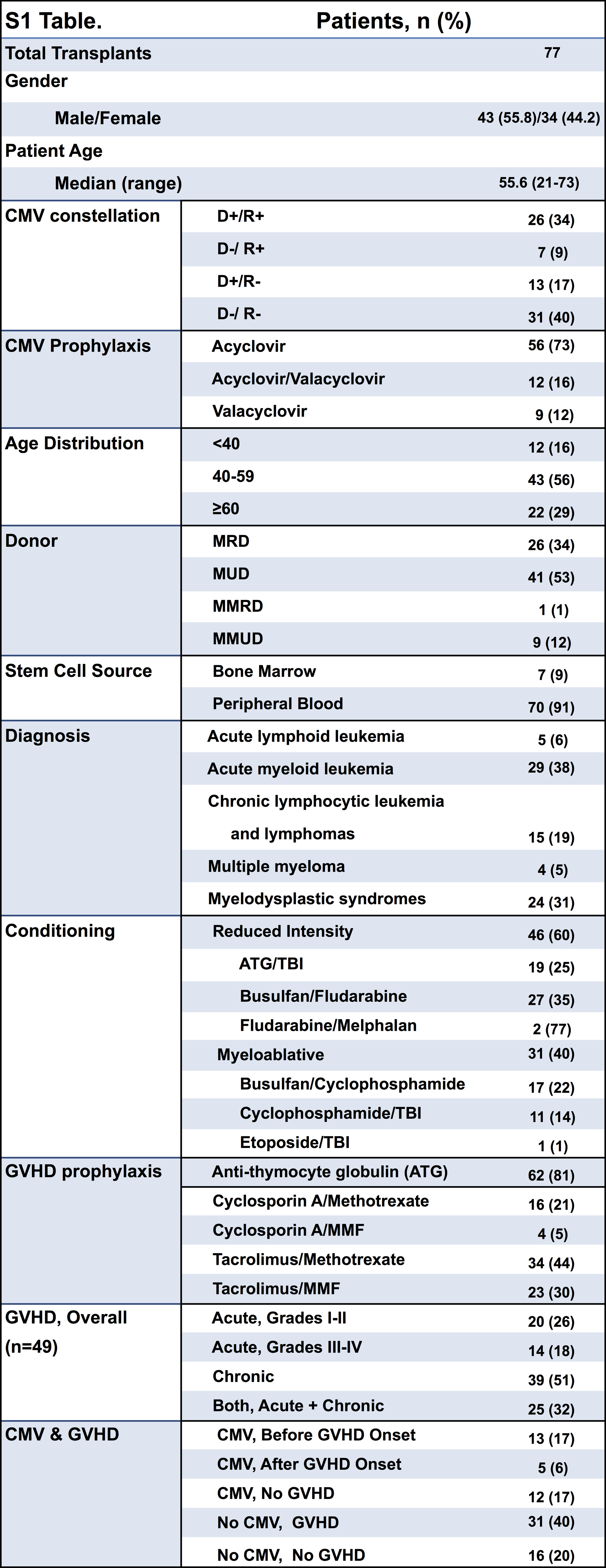

Supplement: S1 Table — (TIF) [file pone.0178763.s011.tif]
